# Dermatological Problems in a Neurology Clinic

**DOI:** 10.7759/cureus.31994

**Published:** 2022-11-28

**Authors:** Anıl Tanburoglu, Sinan Özçelik

**Affiliations:** 1 Neurology, Baskent University Faculty of Medicine, Adana, TUR; 2 Dermatology, Baskent University Faculty of Medicine, Adana, TUR

**Keywords:** intensive care unit, inpatient, neurology, consultation, dermatology

## Abstract

Background: Little is known about the profile of dermatological problems in patients hospitalized in neurology wards or neurological intensive care units (NICUs). In this study, we aimed to provide the demographic and clinical characteristics of inpatients admitted to the neurology ward or the NICU.

Methods: This study was designed as a retrospective observational study. Medical records of patients who consulted with dermatology while they were hospitalized in the neurology ward or the NICU of our hospital, from January 2016 to June 2022, were reviewed retrospectively. Demographic and clinical characteristics of the patients were recorded.

Results: A total of 106 patients, including 86 patients in the ward and 20 patients in the NICU, were included in the study. Forty-nine patients (46.2%) were female and 57 (53.8%) were male. The mean age was 58.47±18.84 years. The frequency of dermatology consultations was 1% overall. The most common causes of hospitalization were ischemic stroke (n=47), demyelinating diseases of the central nervous system (n=10), and encephalitis (n=7). The most common dermatological problems in patients were infectious dermatoses (n=25), drug eruptions (n=18), and physical dermatoses (n=18). While drug eruptions were encountered as a common problem in the neurology ward, physical dermatoses were a common problem in the NICU.

Conclusion: The frequency of dermatology consultations requested from neurology for inpatients was low, especially in the NICU. Drug eruptions in the neurology ward and physical dermatoses in the NICU are encountered as common problems. Neurologists should pay attention to accompanying dermatological problems as well as neurological diseases. Large-scale prospective studies are needed for dermatological problems in patients hospitalized in the neurology clinic.

## Introduction

Various dermatological problems can be observed in patients who are hospitalized in neurology wards or neurological intensive care unit (NICUs). These problems may occur in relation to acute ischemic stroke, intracerebral hemorrhage and neuromuscular diseases, and other causes such as organ failure, sepsis, prolonged bed rest, and side effects of multiple drug treatments - which are particularly common in intensive care units. Due to the foremost characteristics of critically ill patients requiring hospitalization for neurological conditions in wards or intensive care units, dermatological problems are often neglected, or treatment is delayed. Although skin problems are common in patients hospitalized in the neurology clinic, there is a lack of literature on dermatological problems in a neurological setup. In this study, we aimed to determine the demographic and clinical characteristics of the patients in order to draw attention to dermatological problems encountered in neurology wards and intensive care units.

## Materials and methods

Study design

This study was designed as a retrospective observational study. Due to the retrospective design of our study, informed consent was not obtained and was deemed unnecessary by the ethics committee. The study was conducted in accordance with the principles of the Declaration of Helsinki. This study was approved by Baskent University Institutional Review Board and supported by Baskent University Research Fund (approval no: KA22/386).

Inclusion and exclusion criteria

All dermatology consultations requested from the neurology department, from January 2016 to June 2022, were reviewed retrospectively using the hospital automation system. Only patients consulted with the dermatology while they were hospitalized in the neurology ward and the NICU were included in the study. In our hospital, there are three different intensive care units: internal, surgical, and neurological. Only patients in the NICU were included in our study. Outpatient dermatology consultations were excluded from the study.

Data collection

We recorded the demographic characteristics, the need for hospitalization, the length of hospital stays, dermatological diagnoses, the duration of the dermatologic problem, time from onset of complaints to dermatology consultation, and diagnostic procedures used for the dermatological problem (if any) of all patients. Dermatologic problems were categorized into 9 groups. Each group was classified within itself and 40 problems were defined.

Statistical analysis

Statistical analysis was performed on SPSS 20.0 (Statistical Package for Statistical Software for Windows, Armonk, NY). Descriptive analyses for the cases were performed. The results of normally distributed variables were summarized with mean ± standard deviation values, and the results of non-normally distributed variables were summarized with median (min-max) values.

## Results

A total of 10,446 patients were admitted to the neurology ward and NICU of our hospital, from January 2016 to June 2022, and dermatology consultation was requested for 1% (n=106) of these patients. Eighty-six patients (86/6619) in the ward and 20 patients (20/3827) in the NICU were consulted with dermatology (Table 1). Forty-nine patients (46.2%) were female and 57 (53.8%) were male. The mean age of the patients was 58.4 ± 18.8 (min-max: 18-94).

**Table 1:** Distribution of patients in the neurology ward and the neurologic intensive care unit according to neurological diseases CNS; Central Nervous System, CVA; Cerebrovascular Accident

Neurological diseases	Ward	Intensive Care Unit	All patients (%)
Ischemic CVA	34	13	47 (44.34)
Hemorrhagic CVA	2	2	4 (3.77)
Demyelinating diseases of the CNS	10	0	10 (9.43)
Encephalitis	4	3	7 (6.60)
Headache	7	0	7 (6.60)
Epilepsy	6	0	6 (5.66)
Neuropathy	6	0	6 (5.66)
Movement + Gait disorders	5	0	5 (4.72)
Dementia	3	1	4 (3.77)
CNS malignancy	3	0	3 (2.83)
Others	6	1	7 (6.60)
TOTAL	86	20	106

The most common causes of hospitalisation were ischemic cerebrovascular accident (CVA) (n=47), demyelinating diseases of the central nervous system (CNS) (n=10), and encephalitis (n=7). Ischemic CVA was the most common disease in both the ward and the NICU (Table 1). The most common dermatological problems in patients were infectious dermatoses (n=25), drug eruptions (n=18), and physical dermatoses (n=18). Infectious dermatoses were a common problem in patients in both the ward and the NICU (Table 2). While drug eruptions were encountered as a common problem in the neurology ward, physical dermatoses were a common problem in the NICU (Table 2).

**Table 2:** Distribution of patients in the neurology ward and the neurologic intensive care unit according to dermatological problems

Dermatological problems	Ward	Intensive Care Unit	All patients (%)
Infectious Dermatoses	14	11	25 (23.58)
Bacterial	3	2	5 (4.72)
Cellulitis	1	1	2 (1.89)
Folliculitis	1	1	2 (1.89)
Furuncle	1	0	1 (0.94)
Fungal	4	4	8 (7.55)
Dermatophytosis	3	2	5 (4.72)
Candida	1	2	3 (2.83)
Viral	7	3	10 (9.43)
Herpes zoster	5	1	6 (5.66)
Herpes labialis	1	2	3 (2.83)
Verruca	1	0	1 (0.94)
Parasitic	2	0	2 (1.89)
Scabies	2	0	2 (1.89)
Drug eruptions	15	3	18 (16.98)
Maculopapular drug eruption	6	2	8 (7.55)
Urticaria	4	1	5 (4.72)
Acneiform eruption	3	0	3 (2.83)
Papular drug eruption	1	0	1 (0.94)
DRESS	1	0	1 (0.94)
Physical dermatoses	8	10	18 (16.98)
Frictional dermatitis	2	4	6 (5.66)
Decubitus ulcer	3	2	5 (4.72)
Purpura. ecchymosis	1	2	3 (2.83)
Friction blisters	1	2	3 (2.83)
Burn	1	0	1 (0.94)
Eczemas	11	1	12 (11.32)
Seborrheic dermatitis	3	1	4 (3.77)
Contact dermatitis	3	0	3 (2.83)
Asteatotic eczema	2	0	2 (1.89)
Dyshidrotic eczema	2	0	2 (1.89)
Stasis dermatitis	1	0	1 (0.94)
Previous dermatological disease	7	0	7 (6.60)
Psoriasis	4	0	4 (3.77)
Behcet's disease	1	0	1 (0.94)
Bullous pemphigoid	1	0	1 (0.94)
Dermatomyositis	1	0	1 (0.94)
Pruritus	5	2	7 (6.60)
Cutaneous malignancy	2	1	3 (2.83)
Squamous cell carcinoma	2	1	3 (2.83)
Neurocutaneous disease	2	0	2 (1.89)
Neurofibromatosis	2	0	2 (1.89)
Others	14	0	14 (13.21)
Benign skin tumors	4	0	4 (3.77)
Aphthous stomatitis	2	0	2 (1.89)
Prurigo nodularis	2	0	2 (1.89)
Lipodystrophy	1	0	1 (0.94)
Leukocytoclastic vasculitis	1	0	1 (0.94)
Perioral dermatitis	1	0	1 (0.94)
Trichodynia	1	0	1 (0.94)
Erythema nodosum	1	0	1 (0.94)
Lymphedema	1	0	1 (0.94)

The overall median length of hospital stay in the neurology ward and NICU was eight (2-44) days. This value was seven (2-25) days in the ward and 17 (4-44) days in the intensive care unit. The median duration of lesions was 14 (1-360) days, but in 10 patients (9.4%) the time at which the lesion appeared was unknown. The median day of consultation (time from onset of complaints to consultation) was three (1-26) days. It was found that the lesions occurred during hospitalization in a total of 42 (39.6%) patients, 27 of 86 patients in the neurology clinic, and 15 of 20 intensive care patients. A few diagnostic tests were used for dermatological condition, including skin biopsy in seven, Tzanck smear in three, dermatoscopy in two, and mycological examination in two patients.

## Discussion

Dermatology is a medical specialty, which not only covers outpatient settings but also involves inpatient consultations from other departments. In view of the studies that give information about the importance of dermatology consultations in hospital settings, internal medicine had the highest number of all dermatology consultations. Although the neurology department represents an important source of dermatology consultation for inpatients when compared to other departments, the number of consultations can vary. When studies investigating the relationships between clinics in terms of consultations were evaluated, consultation requests originating from the neurology department represented 12%, 9.9%, 9.7%, and 9.8% of all dermatology consultations, as reported by Mancusi et al., Fischer et al., Williams et al., and Ozyurt et al., respectively [1-4].

In our study, the overall dermatology consultation frequency was 1%, while the dermatology consultation rate in the neurology intensive care unit was 0.2%, which shows that the dermatology consultation rate in the neurology intensive care unit was 4 times lower than the rate in the neurology ward. Although we have not found any data in the literature with which we can compare these results specifically, these consultation rates appear to be quite low. However, the frequency of dermatological problems in intensive care patients, in general, was found to be 10.4% by Badia et al., 12% by Pektaş et al., 2.2% by Kara et al., and 1.9% by Lee et al. [5-8]. The relatively low rates of dermatology consultations in our study may be related to the fact that our study only included patients admitted to the NICU, the complicated characteristics of neurocritical patients, and the possible neglect of dermatological problems due to intensive care conditions.

The most common causes of hospitalization were ischemic CVA (n=47), demyelinating diseases of the CNS (n=10), and encephalitis (n=7), which was consistent with the literature. In a study evaluating the profile of patients hospitalized in a neurology clinic, ischemic CVA and demyelinating diseases were the most common neurological diseases [9]. A variety of dermatological problems are encountered in patients of the neurology ward due to the presence of multiple diseases, polypharmacy, and prolonged bed rest. The most common dermatological problems in patients were infectious dermatoses (n=25), drug eruptions (n=18), and physical dermatoses (n=18). Mancusi et al. evaluated patients from all departments who requested dermatology consultation and found that the most common dermatological diagnosis in patients consulted by neurologists was drug eruption, similar to our study [3]. In a study by Williams et al., which included 54 neurological cases, the most common dermatological diagnosis was fungal skin infection, followed by drug eruptions [1]. In our study, drug eruptions and infectious dermatoses were found at a similar rate in the neurology clinic, and fungal skin infections were the most common infectious dermatosis after viral infections. While drug eruptions were mostly seen in consultations requested by the neurology ward, physical dermatoses such as frictional dermatitis/blisters and decubitus ulcers were mostly seen in consultations requested from the NICU. Many factors such as the patients' age, mobility, clinical features, comorbidities, prolonged hospitalization, and impairment of the skin barrier play a role in the development of these lesions, and it is thought that this may be the reason why physical dermatoses are mostly seen in intensive care units [10,11].

The overall median length of stay of patients in the neurology ward or NICU was 8 (2-44) days. This duration was 7 (2-25) days in the ward and 17 (4-44) days in the intensive care unit. Dermatological problems independent of the primary cause of hospitalization may prolong hospitalization in patients [12]. Prolonged hospitalization may increase the likelihood of additional problems and morbidity. The increase in the length of stay, especially in intensive care units, may play a role in the development of pressure ulcers and similar problems due to prolonged bed rest.

In our study, we found that the lesions occurred during hospitalization in 42 (39.6%) patients. The rate of patients who developed a dermatological problem while they were hospitalized was higher in the NICU than in the ward. This may be related to the fact that critically ill patients are at greater risk of developing skin damage than those in the ward. It appears that most of the patients (60.4%) in study had dermatological complaints before hospitalization. The majority of these patients were the patients in the ward. This can be explained by the fact that patients in the neurology ward neglect their dermatological problems or have chronic skin diseases that wax and wane with a flare-up.

The main limitation of our study is its retrospective and single-center design. Another limitation of our study is that it included only consulted cases and the number of cases was relatively low.

## Conclusions

Although various dermatological problems are known to occur in patients hospitalised by the neurology clinics, dermatology consultation rates are low in NICU. Infectious dermatoses are an important problem in neurology inpatients. Drug eruptions in the neurology ward and physical dermatoses in the NICU are encountered as common problems. It is important for neurologists to pay attention to dermatological problems accompanying neurological diseases. We hope that our study will contribute to raising awareness about dermatological problems in patients hospitalised in neurology wards and intensive care units, and we think that large-scale prospective studies are needed on this subject.
